# Impact of a Cooking Intervention on the Cooking Skills of Adult Individuals with Type 2 Diabetes Mellitus: A Pilot Study

**DOI:** 10.3390/nu16111657

**Published:** 2024-05-28

**Authors:** Clarice Mariano Fernandes, Greyce Luci Bernardo, Ana Carolina Fernandes, Ana Paula Gines Geraldo, Daniela Barbieri Hauschild, Débora Kurrle Rieger Venske, Fharlley Lohann Medeiros, Rossana Pacheco da Costa Proença, Paula Lazzarin Uggioni

**Affiliations:** 1 Nutrition in Foodservice Research Centre, Federal University of Santa Catarina, Florianópolis 88040-900, SC, Brazil; clarice.fernandes@posgrad.ufsc.br (C.M.F.); greyce.bernardo@ufsc.br (G.L.B.); ana.fernandes@ufsc.br (A.C.F.); ana.paula.geraldo@ufsc.br (A.P.G.G.); daniela.hauschild@ufsc.br (D.B.H.); debora.venske@ufsc.br (D.K.R.V.); fharlley.lohann@posgrad.ufsc.br (F.L.M.); 2 Nutrition Post-Graduate Program, Federal University of Santa Catarina, Florianópolis 88040-900, SC, Brazil; 3 Nutrition Departament, Federal University of Santa Catarina, Campus Universitário, s/n, Trindade, Florianópolis 88040-900, SC, Brazil

**Keywords:** self-management, practical cooking workshop, healthy eating, food and nutrition education, diabetes education

## Abstract

Objective: To investigate the impact of the Nutrition and Culinary in the Kitchen (NCK) Program on the cooking skills of Brazilian individuals with type 2 diabetes mellitus (T2DM). Methods: A randomized controlled intervention study was performed, with intervention and control groups. The intervention group participated in weekly sessions of the NCK Program for six weeks (including two in-person practical cooking and three online cooking workshops). The cooking recipes were adapted by replacing high glycemic index ingredients with low and medium glycemic index alternatives. Of note, the recipes did not contain added sugars or sweeteners, were based on the use of fresh or minimally processed foods, herbs, and spices, and were sensorially tested by individuals with T2DM before use in the intervention. The study outcomes were participant score changes on the seven scales. A validated online instrument was administered to the control and intervention groups at baseline (T1) and post-intervention (T2). Parametric and non-parametric tests were used to assess the differences between the T1 and T2 parameters. Results: Of the 60 individuals enrolled, 44 answered the questionnaire at both times. The findings revealed a 45.37% ± 93.57% increase in Knowledge of Cooking Terms in the intervention group, whereas the control group showed a 3.82% ± 16.17% reduction (*p* = 0.008). There was an increase in all the other cooking skills and healthy eating scales from T1 to T2 in the intervention group, but the differences were not significant. Conclusions: The development of cooking skills can contribute to increasing culinary knowledge and the availability of time to cook at home. The results contribute to the planning of health actions aimed at individuals with DM2 through culinary interventions and public food and nutrition policies.

## 1. Introduction

Data from the International Diabetes Federation show that in the last two decades, between 2000 and 2021, the number of individuals with diabetes grew by 356%. T2DM is the most prevalent form of the disease, representing more than 90% of all cases of diabetes in the world, and it is associated with greater use of health services and higher rates of hospitalizations due to the increase in the incidence of microvascular and macrovascular disorders [1].

Brazilian and international guidelines recommend healthy eating as a strategy for glycemic control and prevention of complications in T2DM [2,3,4,5]. However, nutrition therapy is a challenging element of the treatment of individuals with T2DM, given that adherence depends on the responsiveness of patients, their family members, and social supports, as well as on psychosocial factors affecting individual self-management [3,6]. Studies conducted in several countries worldwide show that adult individuals with T2DM have less healthy eating habits, such as high consumption of carbohydrates and high glycemic index foods combined with low vegetable consumption [7,8,9].

Diabetes guidelines recommend the development of skills that foster autonomy and self-care [2,3,4]. Studies show that the promotion of cooking skills may contribute to healthier eating [10,11] and assist in diabetes management [12,13,14,15]. In Brazil, official documents also underscore the importance of cooking skills for the development of healthy eating habits [16,17]. Therefore, health promotion through nutritional interventions may be an important strategy for individuals with T2DM [18].

There is limited information on cooking interventions for individuals with T2DM in the scientific literature. Of the studies identified, two used a validated questionnaire to assess the impact of the interventions [19,20] and five were randomized controlled trials [14,15,18,20,21]. None of the cited studies performed interventions that included hands-on cooking classes.

No cooking intervention study focusing on individuals with T2DM in Brazil was identified. A previous intervention study aimed to improve cooking skills in a university community through practical cooking classes and applied an instrument culturally adapted and validated for the Brazilian population to collect the data [22], leading to the creation of the Nutrition and Culinary in the Kitchen (NCK) Program [23,24]. Although the original edition was targeted at university communities without specific pathologies, the NCK program can be adapted to various populations and health conditions, as it is based on guiding principles from official documents on healthy eating [17,25,26,27]. Application of NCK Program interventions is recommended to improve autonomy in the kitchen in other populations, serving as an aid in the treatment and prevention of diseases [23,24].

In view of the encouragement of national and international guidelines to foster self-care, autonomy, and healthier eating habits in individuals with T2DM and recommendations to promote cooking skills through interventions, this study aimed to develop a pilot version of the NCK Program adapted for individuals with T2DM in Brazil and assess its impact on the cooking skills of the participants.

## 2. Materials and Methods

### 2.1. Study Design

This is a controlled, randomized, pilot intervention study conducted in a Brazilian capital city from February to April 2020.

### 2.2. Population and Sample

#### Recruitment

The target population comprised adults with T2DM with no associated complications, such as retinopathy or nephropathy, residing in a metropolitan region encompassing 22 municipalities in Brazil [28].

The sample size was calculated considering an average difference of 35% in the outcome of the Knowledge of Cooking Terms and Techniques (CTT) scale, based on the study by Bernardo et al. [24]. Other parameters were a study power of 80%, margin of error of 5%, and confidence level of 95%, resulting in a sample of 21 individuals with T2DM per group. Random losses of 20% and 10% were applied to account for confounding factors [29], affording a minimum required sample of 30 individuals for the intervention (IG) and control (CG) groups, totaling 60 individuals.

To participate in the research, individuals had to meet the following inclusion criteria: (i) have a diagnosis of T2DM, (ii) be over 18 years of age, (iii) have availability to participate in the cooking classes, and (iv) sign the informed consent form. The exclusion criteria were as follows: type 1 diabetes mellitus, gestational diabetes, diabetes-associated complications, or any health condition precluding participation in cooking classes. 

Recent biochemical information, such as the Hba1c or general glucose profile, was not established as an inclusion criterion in the research, as the change in these parameters is not part of the main objective. To respond to criterion (i), individuals were asked to present a biochemical exam, whether recent or not. Likewise, the glycemic control strategy was not questioned, but changes in dietary patterns were assessed using the investigation instrument.

The intervention was promoted through electronic media and physical posters containing a link and QR code to an online application form. The posters were displayed at strategic points crossed by large numbers of people in the metropolitan area of the city (university hospital and emergency care units). Participants were recruited between August 2019 and January 2020.

### 2.3. Randomized Allocation

This study used a convenience sample of voluntary participants who were included in the study until the sample size was reached. The first 60 individuals who agreed to participate were randomly allocated to one of the two groups (IG or CG) using the online tool Research Randomizer^®^ (http://www.randomizer.org, accessed on 15 February 2020).

### 2.4. Cooking Intervention

The NCK Program consists of five hands-on cooking classes and a food purchase and selection workshop at a store that sells fresh produce and packaged foods. Classes were held weekly for six weeks, and each meeting lasted 3 h, totaling 18 h of intervention [23,24].

The cooking recipes from the NCK Program intervention [23,24] are based on healthy eating recommendations [17,30,31,32,33], valuing fresh foods, herbs and spices as seasonings, healthier cooking techniques (e.g., baking, sautéing, and steaming), seasonal and organic fruits and vegetables, and elimination of ultra-processed foods and ingredients containing industrially produced trans fats.

The intervention consisted of the pilot test of the NCK Program adapted for individuals with T2DM. The cooking recipes (Table 1) were adapted by replacing high glycemic index ingredients with low and medium glycemic index alternatives. Of note, the recipes did not contain added sugars or sweeteners and were sensorially tested by individuals with T2DM before use in the intervention [34].

Initially, the intervention had been planned to be carried out entirely in person, like the original program. However, after the first three in-person cooking classes, it was necessary to change the program to an online format because of the public health emergency of the coronavirus disease 2019 (COVID-19) pandemic [35]. Therefore, the last three classes were video-recorded as demonstrative online classes by the main investigator (CMF) and made available to the participants through a video-sharing website. The class objectives and cooking recipes in the demonstrative classes followed the initial methodological proposal (Table 1). 

### 2.5. In-Person Cooking Classes

The IG individuals participated in the NCK Program intervention for six weeks distributed over a period of two months, including in-person classes or online classes according to the weekly schedule. In line with the NCK Program scheme (Table 1), each class had a specific objective, and the topics learned in previous classes were reinforced by practical application in the following meetings. 

The in-person sessions included various demonstrations of ingredients, recipes, and cooking techniques, as well as discussions on nutrition, recipe preparation, and overall impressions at the end of the class. The IG participants were divided into groups and allocated to workbenches to prepare the recipes. The in-person sessions were led by 10 facilitators (undergraduate and graduate nutrition students) divided into groups of 2 or 3 per workbench. The facilitators assisted the participants in putting into practice what had been presented in class. All the facilitators received 3.5 h of theoretical and practical training prior to the beginning of the cooking classes. 

The food purchase and selection workshop was conducted in-person at a store that sells fresh fruits, vegetables, fish, and bread at affordable prices. 

### 2.6. Demonstrative Online Classes

Video recordings of the weekly cooking classes were produced and added to a video-sharing platform. Each video comprised demonstrations of cooking recipes (3 to 4 min per preparation) and a discussion on cooking techniques and recipes (9 min per class). In the video classes, the main researcher carried out all the steps. The participants were encouraged to try them at home and send pictures and videos of their recipes to a group chat on a multiplatform instant messaging application for smartphones, which was also used to resolve doubts regarding the video classes. Pictures and videos shared by the participants were used to confirm participation in each online class.

### 2.7. Control Conditions

During the entire intervention period, the CG participants were not contacted. The participants were initially informed that they would be invited to participate in a practical cooking class directed toward T2DM after completing the assessment instrument to measure their cooking skills and healthy eating at baseline (T1) and post-intervention (T2). However, because of social distancing measures brought about by the COVID-19 pandemic, it was not possible to carry out face-to-face classes as initially planned. Therefore, at the end of the study, the CG individuals received a booklet containing guidelines on self-care and healthy eating for DM management, in addition to cooking recipes based on low glycemic index ingredients.

### 2.8. Data Collection

The participants were evaluated at T1 and T2. The IG participants provided personal information and completed the assessment instrument before the first face-to-face class (T1) in a computer laboratory. At T2, the participants received a link via an electronic message to complete the assessment instrument. For those participants who found it difficult to complete the questionnaire, the main researcher provided assistance through telephone calls. Also at T1 and T2, the CG participants received a link to the online survey via electronic message or telephone. Individuals took 40 min on average to complete the instrument.

### 2.9. Instrument for Assessing Cooking Skills and Healthy Eating

The instrument used to measure the outcomes of this research was culturally adapted and validated for the Brazilian population [22,34]. It includes items on cooking skills and healthy eating distributed in eight sections. For each section, the sum (yes/no answers) or mean (scales) of the items was calculated at each time of instrument application (T1 and T2). Below, we describe what each section evaluated and how they were scored.

1. Accessibility and Availability of Fruits and Vegetables (AAFV): at-home availability of fruits and vegetables over the previous week; the section is composed of 8 items with yes or no questions, scored as 1 or 0, respectively.
2. Cooking Attitude (CA): how respondents feel about cooking; composed of 7 items scored on a 5-point Likert scale ranging from “strongly disagree” to “strongly agree”.
3. Cooking Behavior at Home (CBH): frequency of common cooking activities at home; composed of 6 items scored on a 5-point Likert scale (1 = not at all, 2 = one to two times a month, 3 = once a week, 4 = several times a week, and 5 = almost every day).
4. Cooking Behavior Away from Home (CBAH): frequency of common cooking activities away from home; composed of 5 items scored on a 5-point Likert scale (1 = not at all, 2 = one to two times a month, 3 = once a week, 4 = several times a week, and 5 = almost every day).
5. Self-Efficacy for Using Basic Cooking Techniques (SECT): degree of confidence in performing basic cooking techniques; composed of 18 items scored on a 5-point Likert scale ranging from “not confident at all” to “extremely confident”.
6. Self-Efficacy for Using Fruits, Vegetables, and Seasonings (SEFVS): degree of confidence in using fruits and vegetables when cooking; composed of 9 items scored on a 5-point Likert scale ranging from “not confident at all” to “extremely confident”.
7. Produce Consumption Self-Efficacy (SEPC): degree of confidence in meeting the government’s recommendations for fruit and vegetable consumption; composed of 3 items scored on a 5-point Likert scale ranging from “not confident at all” to “extremely confident”.
8. Knowledge of Cooking Terms and Techniques (CTT): level of cooking knowledge; composed of 8 items with multiple choice answers (each correct answer scored as 1 point).

For the sample characterization, we collected sociodemographic and personal data, including age, sex, date and value of the last glycated hemoglobin test, time since diagnosis of T2DM, living arrangement (living alone or with friends, family, or parents), and cooking characteristics (daily time available to cook, equipment and utensils available at home, self-reported cooking knowledge, source of cooking experience, and place of main meal). The self-reported height and weight were used for the body mass index (BMI) calculation.

### 2.10. Data Analysis

The sociodemographic and personal characteristics were analyzed by descriptive statistics, using the mean and standard deviation for symmetric or median numerical variables and the interquartile range (IQR) for asymmetric numerical variables. The symmetry of the continuous numerical variables was measured using the coefficient of variation and histograms. For the categorical variables, data were presented as absolute and relative frequencies. 

The BMI values were used to categorize individuals as having normal weight or excess weight (overweight and obesity) according to the parameters of the World Health Organization [36]. To assess the differences between groups at baseline, we analyzed data using chi-square or Fisher’s chi-square tests for categorical variables, and the *t*-test or Mann–Whitney *U*-test, depending on the data symmetry. Following confirmation of the symmetry of the outcome variables through the coefficient of variability, the paired *t*-test was employed or intragroup evaluation of the outcomes over the two periods (T1 and T2). A paired *t*-test was used for intragroup evaluation of the outcomes over the two periods (T1 and T2). To compare the changes between groups from T2 to T1, we applied the *t*-test. Analyses were performed considering differences in the means, and tests were chosen on the basis of the distribution symmetry of the variables. It should be noted that, after the initial analysis, nonparametric tests were also performed to assess the consistency of the results, which were similar to those found in the previous analysis. The analyses were performed using Stata^®^ version 13.0 (StataCorp, College Station, TX, USA), and the significance level was set at *p* < 0.05. 

### 2.11. Ethical Aspects

Ethical approval was obtained from the Human Research Ethics Committee of the Federal University of Santa Catarina (protocol number 1,318,443), which is in accordance with the ethical standards of the 1964 Declaration of Helsinki. Informed consent was obtained from all the participating individuals at the first cooking workshop. The participants did not receive gifts or incentives for their participation. 

## 3. Results

### 3.1. Follow-Up and Sample Losses

**Figure 1:**
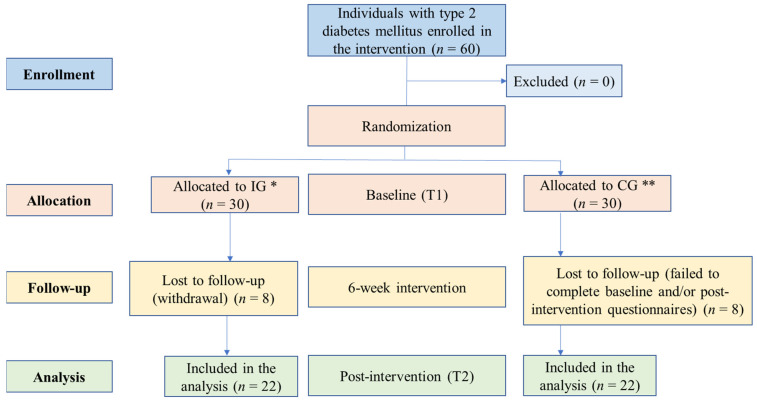
Flow diagram of participants. * Intervention group. ** Control group.

Of the 60 individuals who agreed to participate in the study, 44 remained until the end of the study and completed the instrument at T1 and T2, resulting in a loss of 26.6% (*n* = 16). The COVID-19 pandemic affected the routine of some IG participants, such as a change of residence for better care and monitoring. Thus, some participants encountered difficulties in accessing the internet or were not able to cook. Anxiety related to the pandemic and family members diagnosed with COVID-19 were reported, which might have contributed to the withdrawal of some participants. The participant flow chart, including losses during the study, is shown in Figure 1.

### 3.2. Characteristics of Participants at Baseline

Table 2 presents the characteristics of the participants per group at baseline. No significant differences were found between the CG and the IG. On average, 5.3 ± 0.8 of the 6 sessions were completed by the IG members.

In both groups, most participants lived with their partner (40.9%), partner and child (20.5%), or alone (15.9%). Of the 44 individuals participating in the research (IG and CG), 93.2% reported knowing how to cook, and such knowledge was reported to have been mainly acquired from family members (61.3%), alone (45.5%), or the internet (18.2%). The cooking skills and healthy eating scales did not differ significantly between the IG and the CG at baseline. 

### 3.3. Intragroup Changes in Cooking Skills and Healthy Eating

In the IG, the means of all the cooking skills and healthy eating scales were numerically higher at T1 than at T2; however, the increase was only significant for CTT (*p* = 0.017). In the CG, the means of six of the eight scales were numerically lower at T1 than at T2, but the differences were not significant (Table 3).

At T2, there was an increase in the median time available for cooking in the IG (155 min at T1 to 180 min at T2; p25–p75 = 120–240, *p* = 0.867) compared with the CG (165 min at T1 to 120 min at T2; p25–p75 = 90–240). In the CG, the time available for cooking decreased over time, *p* = 0.867).

### 3.4. Changes in Cooking Skills and Healthy Eating between Groups

**Figure 2:**
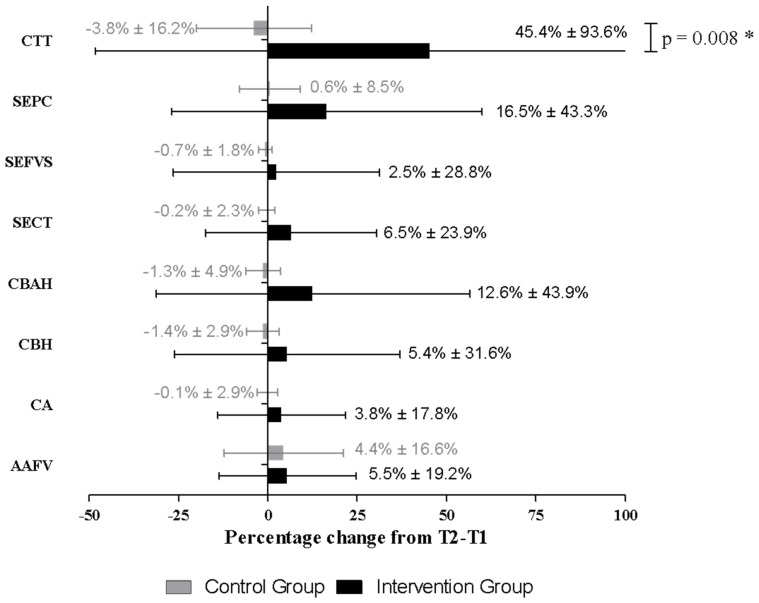
Percentage change from baseline in cooking skill scores of control and intervention groups of individuals with type 2 diabetes mellitus. Bars represent the mean, and error bars represent the standard deviation. AAFV, Accessibility and Availability of Fruits and Vegetables; CA, Cooking Attitude; CBH, Cooking Behavior at Home; CBAH, Cooking Behavior Away from Home; SECT, Self-Efficacy for Using Basic Cooking Techniques; SEFVS, Self-Efficacy for Using Fruits, Vegetables, and Seasonings; SEPC, Produce Consumption Self-Efficacy; CTT, Knowledge of Cooking Terms and Techniques. * *p* = 0.008 (Significance diference between groups).

In comparing the differences between the groups at T2 and T1, we found that CTT was higher (*p* < 0.008) in the IG (0.95 ± 1.73) than in the CG (0.27 ± 0.23) (Figure 2). The CTT scores increased by 45.37% ± 93.57% in the IG and decreased by 3.82% ± 16.17% in the CG (*p* = 0.008). There were no other significant differences between the groups in the percentage change of the scale scores from T1 to T2 (Table 3).

## 4. Discussion

The results of this study demonstrated an increase in CTT from baseline (T1) to follow-up (T2) among the IG participants, as well as higher CTT scores in the IG than in the CG. Although there were no significant changes in the other cooking ability and healthy eating scales in the IG, the scales showed an increasing trend. In the CG, however, there was a decreasing trend in six (CA, CBH, CBAH, SEPC, SECT, and CTT) of the eight scales between T1 and T2. Thus, the NCK Program contributed to maintaining cooking skills among individuals with T2DM, regardless of the initial score for each scale.

At T2, the IG had a median increase of 60 min in the time available for cooking compared with the CG. The findings demonstrate a positive impact of the program on this variable in the target population. The results corroborate those of Dasgupta et al. [18], who identified an increase of 30 min in the time spent on meal preparation among 53 Canadian individuals with T2DM after 15 theoretical and practical classes on eating habits and meal preparation. 

In the present study, the increase in all the self-efficacy scales (SEPC, SECT, SEFVS), although not significant, may indicate an increase in confidence in performing basic cooking techniques (SECT), meeting official recommendations for fruit and vegetable consumption (SEPC), and using fruits, vegetables, and spices during cooking (SEFVS). Dasgupta et al. [18] found a significant increase in confidence in using cooking skills among individuals with T2DM participating in cooking interventions. According to Lavelle et al. [37,38], confidence in cooking is associated with a better diet quality, such as increased consumption of fruits and vegetables and greater use of fresh and minimally processed ingredients.

The nonsignificant increase in CA in the IG compared with the CG was in agreement with the findings of Monlezun et al. [20]. The authors carried out a randomized controlled study with individuals with T2DM participating in six theoretical–practical cooking classes. The cited study identified an increase, albeit not significant, in positive attitudes toward meeting healthy eating recommendations. These findings may suggest that cooking intervention programs have the potential to stimulate necessary changes in the eating behavior of individuals with T2DM. Of note, the small sample size might have influenced our results; thus, it is recommended to carry out cooking interventions with a more representative population.

Although there is no consensus on the topic, some studies have reported that individuals with T2DM have low adherence to dietary interventions [39,40], which seems to be related to psychological, psychosocial, and sociocultural conditions [39,41]. In the present study, this factor might have represented a barrier to participants’ adherence to the program, thereby contributing to the non-significance of the results.

It should be noted that when the NCK Program was applied to university students, the cooking classes were held in-person [24]. In this study, because of the social restrictions brought about by the COVID-19 pandemic, it was necessary to perform the last three classes remotely, as explained in the Methods section. Therefore, three face-to-face meetings and three video classes were applied. The remote nature of the latter classes, combined with the possible difficulties faced by individuals at the beginning of the pandemic, might have influenced the impact of the NCK Program on the eating behavior of the participants.

Recent studies showed that the beginning of social distancing caused changes in food consumption worldwide; there was a decrease in fruit and vegetable consumption and an increase in that of ultra-processed foods, such as chocolate, snacks, and frozen foods [42,43]. A higher intake of carbohydrates by individuals with T2DM was also reported [44]. These factors might have impacted the results of the intervention, given that the COVID-19 pandemic generated changes not only in the routine of individuals but also in food purchase and consumption [43,45] and food insecure [46].

It is important to highlight that the adaptation of the NCK Program cooking recipes to individuals with T2DM [34] included the replacement of all high glycemic index ingredients with low and medium glycemic index ingredients, as well as the elimination of added sugars and sweeteners, in line with the recommendations of the American Diabetes Association [4]. The stimulus to prepare meals with a low glycemic index during the intervention might have positively contributed to the participants’ sense of self-care and disease management, promoting eating habits that contribute to glycemic control. A low glycemic load and carbohydrate-restricted diets seem to be effective in reducing blood glucose, insulin and the need for medications [47,48]. 

This study was pioneering in developing a cooking skills intervention for individuals with T2DM and using a validated instrument to assess the impact of the intervention. We also highlight the uniqueness of the intervention in using cooking recipes adapted to individuals with T2DM. The recipes had a low glycemic index, no added sugars or sweeteners, and a controlled salt content, and they were based on the use of fresh or minimally processed foods, herbs, and spices. The meals were previously tested and approved by individuals with T2DM.

An online culinary intervention, consisting of eight culinary workshops (four of these workshops were theoretical sessions and the other four consisted of cooking online classes), carried out during the pandemic period, was able to improve the eating and cooking habits of individuals with T2DM [21]. However, unlike the present study, culinary preparations with low and medium glycemic index ingredients were not considered for use in the cooking program.

Some researchers observed that individuals seem to have spent more time cooking during the pandemic [43,49]. In the present study, however, only individuals participating in the NCK Program reported an increase in the time available for cooking, confirming the hypothesis of the program’s positive impact. A greater amount of time spent preparing meals seems to be related to the use of basic and fresh ingredients. Lavelle et al. [38] and Silver et al. [49] argued that the time spent cooking may also be associated with a lack of cooking knowledge and practice, not only with the type of food prepared. 

Despite the obstacles related to adherence to dietary recommendations by individuals with T2DM, changes in the form of workshop application, and changes in dietary patterns during the pandemic, the increase in the CTT scores among the IG individuals demonstrated the positive impact of the NCK Program on the acquisition of knowledge related to cooking and healthy eating. It is also noteworthy that 93.2% of the sample reported knowing how to cook; however, it was not possible to evaluate the ingredients and techniques used by the participants at baseline. Regardless of the level of self-reported cooking skill at baseline, the intervention promoted an increase in knowledge about cooking terms and techniques, one of the dimensions of cooking skills. Such knowledge is important for the development of autonomy in meal preparation, as recommended by diabetes guidelines [2,3,4].

Some limitations of the present study should be considered in future interventions. Despite withdrawal leading to a reduction in the sample size, the number of participants who withdrew was equivalent in both groups. Furthermore, while the sample size may present limitations to the study’s conclusions, a significant difference was identified in one of the scales, thereby aiding in hypothesis generation and providing direction for future research. Individuals were allowed to choose whether to participate or not, possibly resulting in self-selection bias [50]. This type of bias is implicit in intervention studies and may decrease the external validity, without, however, affecting internal validity. The use of adequate methods for randomization can contribute to minimizing this type of bias [51].

The need to complete the questionnaire at T1 and T2 might have represented a limitation for individuals who are not used to filling out online forms. However, assistance was provided at the time of instrument application via multiplatform instant messaging and telephone calls.

Because of the COVID-19 pandemic, it was necessary to interrupt the face-to-face practical classes halfway through the program and replace them with remote meetings. However, the methodology of the workshops was adapted for the demonstrative video classes, which have been shown to promote healthy eating [52,53] and diabetes mellitus management [54]. The availability of online cooking classes provided autonomy and encouragement for some participants, who sent photos of the meals they prepared during the workshops. For others, however, the lack of practical stimulation might have limited the knowledge acquisition. Other factors related to the pandemic, such as difficulty in acquiring food and changes in eating and lifestyle habits, may also have influenced the preparation of the cooking recipes.

Finally, an important strength of this study was the use of rigorous methods to conduct the interventions, with the randomization of participating groups and use of an instrument adapted and validated for the Brazilian population.

Similar studies should be carried out with individuals with other non-communicable diseases. Future studies should carry out qualitative research with individuals with T2DM to identify their perceptions about the treatment of the disease and the impact of the cooking workshops on other subjective issues, such as the difficulty of following dietary recommendations. It is suggested to apply a fully practical methodology and evaluate the sustained (long-term) effects of the NCK Program for T2DM, including in relation to other health-related outcomes, such as serum markers and body composition.

## 5. Conclusions

It was observed that participation in the NCK Program led to a significant increase in the CTT scores of individuals with T2DM. This conclusion may suggest that, even during confinement due to the COVID-19 pandemic, the development of culinary skills can contribute to increased culinary knowledge and greater availability of time to cook at home.

The results of the present study may assist in the implementation of future practical or theoretical–practical and face-to-face culinary interventions for individuals with T2DM, aiming to promote healthier eating habits and self-management of the disease. The use of culinary recipes without added sugar or sweeteners, adapted with low and medium glycemic index alternatives, represents a strong point of this study for future interventions in populations with T2DM to promote eating habits that contribute to glycemic control.

The findings may contribute to the planning of public food and nutrition policies that include interventions focused on promoting culinary skills and healthy eating among individuals with other non-communicable diseases.

## Figures and Tables

**Table 1:** Nutrition and Culinary in the Kitchen Program for individuals with type 2 diabetes mellitus adapted to the context of the COVID-19 pandemic.

Cooking Classes	Modality	Objectives	Cooking Techniques ^a^ and Recipes
1	In-person (practical)	Learn basic cooking techniques and skills to prepare a pleasant, healthy, and easy meal	(1) Roasted vegetables (2) Fruit salad with flavored water (3) Omelet (4) Homemade vegetable and chicken broth ^a^ (5) Sautéed, roasted, and pressure-cooked chicken ^a^
2	In-person (practical)	Learn about the importance of including fruits and vegetables in the daily diet	(1) Chicken salad (2) Yogurt salad dressing ^a^ (3) Oatmeal pan bread ^a^ (4) Creamy avocado sorbet -Vegetable blanching ^a^ -Cleaning and storing leafy greens ^a^
3	In-person (practical)	Visit a fresh food market, learn to select produce and understand food nutrition labeling ^b^	-How to choose a weekly selection of fresh produce, especially fruits and vegetables, at affordable prices -Discussion about food nutrition labeling
4	Remote (demonstrative online lesson)	Learn to prepare a healthy and complete meal using pantry items	(1) Chopped cauliflower with garlic (2) Mixed salad (3) Steak with onions ^a^ (4) Black beans and chayote ^a^ (5) Vinaigrette ^a^ (6) Fresh apple
5	Remote (demonstrative online lesson)	Learn about the importance of eating whole grains and taking flavors into account in meal planning	(1) Roasted meatballs (2) Broccoli, lentil, and peanut salad (3) Homemade tomato sauce ^a^ (4) Grilled zucchini slices (5) Lime salad dressing (6) Fruit platter (7) Seasoned salt ^a^
6	Remote (demonstrative online lesson)	Apply cooking skills learned in class to prepare a full meal	(1) *Moqueca* ^c^ (2) White bean salad (3) Spearmint and basil salad dressing (4) Sugar- and sweetener-free oatmeal and fruit cake (5) Fish *pirão* ^a,d^ (6) Butter-fried *farofa* ^a,e^ with collard greens and eggs

^a^ Presentation of cooking recipes and practical demonstrations. After class, the participants put the learned content into practice. ^b^ Food purchase and selection workshop. ^c^ *Moqueca* is a traditional Brazilian dish of fish stew with coconut milk. ^d^ *Pirão* is a traditional Brazilian dish of cassava flour cooked in a hot broth. ^e^ *Farofa* is a traditional Brazilian dish of cassava flour fried in vegetable oil or butter, which may be supplemented with other ingredients, such as vegetables, egg, and meat.

**Table 2:** Sociodemographic characteristics and cooking skills of individuals with type 2 diabetes mellitus participating in the Nutrition and Culinary in the Kitchen Program at baseline (*n* = 44).

Characteristic	Total (*n* = 44)	Control Group (*n* = 22)	Intervention Group (*n* = 22)	*p*-Value *
Sex, *n* (%)				
Male	16 (36.4)	7 (31.8)	9 (40.9)	0.531 ^a^
Female	28 (63.6)	15 (68.2)	13 (59.1)	
Age (years)				
Mean (±SD)	58.9 ± 10.3	59.8 ± 11.6	59.8 ± 9.15	1.000 ^b^
Body mass index (kg/m^2^)				
Mean (±SD)	29.3 ± 4.9	29.8 ± 5.4	28.8 ± 4.4	0.504 ^b^
Body mass index, *n* (%)				
Non-overweight	11 (25.0)	6 (27.3)	5 (22.7)	0.728 ^a^
Overweight	33 (75.0)	16 (72.7)	17 (77.3)	
Do you know how to cook? *n* (%)				
Yes	41 (93.2)	20 (90.9)	21 (95.5)	1.000 ^c^
No	3 (6.8)	2 (9.1)	1 (4.5)	
Where do you usually have your main meal? *n* (%)				
At home	37 (84.1)	20 (90.9)	17 (77.3)	0.412 ^c^
Away from home	7 (15.9)	2 (9.1)	5 (22.7)	
Duration of diabetes (years)				
Median [p25, p75]	8.5 [3.00, 13.00]	7.5 [3.00, 13.00]	10.5 [3.00, 13.00]	0.183 ^d^
Daily time spent cooking (min)				
Median [p25, p75]	120 [85, 180]	120 [60, 240]	120 [90, 180]	0.867 ^d^
Cooking skills ^e^, mean (±SD)				
AAFV	6.73 ± 1.39	6.68 ± 1.21	6.77 ± 1.41	0.819 ^b^
CA	4.10 ± 0.62	4.14 ± 0.60	4.05 ± 0.65	0.682 ^b^
CBH	3.43 ± 0.68	3.46 ± 0.74	3.36 ± 0.63	0.638 ^b^
CBAH	1.96 ± 0.75	2.07 ± 0.80	1.85 ± 0.71	0.344 ^b^
SECT	3.79 ± 0.68	3.76 ± 0.74	3.82 ± 0.63	0.772 ^b^
SEFVS	3.76 ± 0.84	3.86 ± 0.71	3.67 ± 0.95	0.443 ^b^
SEPC	3.71 ± 0.63	3.73 ± 0.45	3.69 ± 0.77	0.854 ^b^
CTT	4.68 ± 2.08	4.50 ± 2.20	4.86 ± 1.98	0.568 ^b^

^a^ Chi-square test. ^b^ *t*-test. ^c^ Fisher’s chi-square test. ^d^ Mann–Whitney test. ^e^ AAFV, Accessibility and Availability of Fruits and Vegetables; CA, Cooking Attitude; CBH, Cooking Behavior at Home; CBAH, Cooking Behavior Away from Home; SECT, Self-Efficacy for Using Basic Cooking Techniques; SEFVS, Self-Efficacy for Using Fruits, Vegetables, and Seasonings; SEPC, Produce Consumption Self-Efficacy; CTT, Knowledge of Cooking Terms and Techniques. * Statistical significance was set at *p* ≤ 0.05.

**Table 3:** Comparison of cooking skill and diet quality scores between control and intervention groups of individuals with type 2 diabetes mellitus at baseline (T1) and post-intervention (T2).

Scale ^a,b^	Intervention Group			Control Group		
	T1	T2	*p*-Value *	T1	T2	*p*-Value *
AAFV	6.77 ± 1.41	7.00 ± 1.31	0.329	6.68 ± 1.21	6.95 ± 1.40	0.162
CA	4.05 ± 0.65	4.13 ± 0.52	0.584	4.14 ± 0.60	4.12 ± 0.57	0.576
CBH	3.36 ± 0.63	3.39 ± 0.60	0.865	3.46 ± 0.74	3.40 ± 0.67	0.176
CBAH	1.85 ± 0.71	2.03 ± 0.94	0.279	2.07 ± 0.80	2.04 ± 0.80	0.185
SECT	3.82 ± 0.63	3.88 ± 0.94	0.754	3.76 ± 0.74	3.73 ± 0.74	0.096
SEFVS	3.67 ± 0.95	3.92 ± 0.71	0.279	3.86 ± 0.71	3.88 ± 0.74	0.833
SEPC	3.69 ± 0.77	3.83 ± 0.78	0.406	3.73 ± 0.45	3.72 ± 0.44	0.584
CTT	4.86 ± 1.98	5.81 ± 1.40	0.017	4.50 ± 2.20	4.23 ± 2.14	0.266

^a^ AAFV, Accessibility and Availability of Fruits and Vegetables; CA, Cooking Attitude; CBH, Cooking Behavior at Home; CBAH, Cooking Behavior Away from Home; SECT, Self-Efficacy for Using Basic Cooking Techniques; SEFVS, Self-Efficacy for Using Fruits, Vegetables, and Seasonings; SEPC, Produce Consumption Self-Efficacy; CTT, Knowledge of Cooking Terms and Techniques. ^b^ Data are presented as the mean ± standard deviation. * Paired *t*-test (*p* ≤ 0.05).

## Data Availability

The data presented in this study are available on request from the corresponding author.
